# Anaplastic Lymphoma Kinase Gene Copy Number Gain in Inflammatory Breast Cancer (IBC): Prevalence, Clinicopathologic Features and Prognostic Implication

**DOI:** 10.1371/journal.pone.0120320

**Published:** 2015-03-24

**Authors:** Min Hwan Kim, Soohyeon Lee, Ja Seung Koo, Kyung Hae Jung, In Hae Park, Joon Jeong, Seung Il Kim, Seho Park, Hyung Seok Park, Byeong-Woo Park, Joo-Hang Kim, Joohyuk Sohn

**Affiliations:** 1 Division of Medical Oncology, Department of Internal Medicine, Yonsei University College of Medicine, Seoul, Korea; 2 Department of Pathology, Yonsei University College of Medicine, Seoul, Korea; 3 Department of Oncology, Asan Medical Center, University of Ulsan College of Medicine, Seoul, Korea; 4 Center for Breast Cancer, National Cancer Center, Goyang, Korea; 5 Department of Surgery, Gangnam Severance Hospital, Yonsei University College of Medicine, Seoul, Korea; 6 Department of Surgery, Yonsei University College of Medicine, Seoul, Korea; 7 Department of Internal Medicine, Yonsei University College of Medicine, Seoul, Korea; University of North Carolina School of Medicine, UNITED STATES

## Abstract

**Background:**

Inflammatory breast cancer (IBC) is the most aggressive form of breast cancer, and its molecular pathogenesis still remains to be elucidated. This study aimed to evaluate the prevalence and implication of anaplastic lymphoma kinase (ALK) copy number change in IBC patients.

**Methods:**

We retrospectively collected formalin-fixed, paraffin-embedded tumor tissues and medical records of IBC patients from several institutes in Korea. ALK gene copy number change and rearrangement were assessed by fluorescence *in situ* hybridization (FISH) assay, and ALK expression status was evaluated by immunohistochemical (IHC) staining.

**Results:**

Thirty-six IBC patients including those with HER2 (+) breast cancer (16/36, 44.4%) and triple-negative breast cancer (13/36, 36.1%) were enrolled in this study. ALK copy number gain (CNG) was observed in 47.2% (17/36) of patients, including one patient who harbored ALK gene amplification. ALK CNG (+) patients showed significantly worse overall survival compared to ALK CNG (-) patients in univariate analysis (24.9 months vs. 38.1 months, *p* = 0.033). Recurrence free survival (RFS) after curative mastectomy was also significantly shorter in ALK CNG (+) patients than in ALK CNG (-) patients (n = 22, 12.7 months vs. 43.3 months, *p* = 0.016). Multivariate Cox regression analysis with adjustment for HER2 and ER statuses showed significantly poorer RFS for ALK CNG (+) patients (HR 5.63, 95% CI 1.11–28.44, *p* = 0.037).

**Conclusion:**

This study shows a significant presence of ALK CNG in IBC patients, and ALK CNG was associated with significantly poorer RFS.

## Introduction

Inflammatory breast cancer (IBC) is the most aggressive form of breast cancer, characterized by erythematous and edematous changes of the involved breast with numerous dermal tumor emboli and lymphatic dilatation upon microscopic examination. IBC comprises 1%–6% of all breast cancers, and the distinct clinicopathological features and dismal prognosis distinguish IBC from locally advanced non-IBC. The 5-year survival rate of IBC patients is about 40% despite recent advances in multimodal treatment including chemotherapy, surgery, and radiotherapy [1,2], and the median recurrence free survival (RFS) and the median overall survival (OS) of IBC are 2.3 years and 4.2 years, respectively, according to a previous report [3]. Considering that IBC is almost always found to be an advanced disease, effective systemic therapy is imperative. However, a specific targeted therapy that could improve treatment outcome of IBC patients is yet to be developed. The majority of IBCs are hormone receptor-negative, and the proportion of HER2-positive and triple-negative breast cancer (TNBC) cases is higher in IBC than in non-IBC. Although several genes such as RHO-C GTPase, p53, and WISP3 have been shown to be altered in IBC tumors [4–7], the molecular pathogenesis and target identification of IBC still needs to be elucidated [8].

Anaplastic lymphoma kinase (ALK) is a receptor tyrosine kinase that has been regarded as a valuable molecular target following the success of a cMet and ALK inhibitor, crizotinib, in non-small cell lung cancer (NSCLC) tumors with ALK gene rearrangement [9,10]. ALK rearrangement and mutation are involved in the pathogenesis of human malignancies such as anaplastic lymphoma, neuroblastoma, and myofibroblastic tumor. In addition to ALK gene rearrangement, ALK gene copy number gain (CNG) and amplification have also been reported in NSCLC [11], colorectal carcinoma [12], renal cell carcinoma (RCC) [13], rhabdomyosarcoma [14], and neuroblastoma tumor [15], but their biological significance and relation to ALK inhibitor susceptibility remain uncertain.

Recent studies have reported that there are considerable CNGs and amplifications in IBC cell lines and IBC patient tumors [16–18]. Furthermore, a preclinical study showed that ALK-amplified tumor cells isolated from IBC patients were highly sensitive to the anti-proliferative effect of crizotinib, but resistant to paclitaxel [16]. Based on these reports, ALK CNG and amplification are potentially druggable genetic alterations in IBCs, and validation of their clinical implications and prognostic relevance is warranted. This study aimed to evaluate the prevalence and implication of ALK copy number changes in IBC patients. We used formalin-fixed paraffin-embedded (FFPE) tumors to investigate protein expression levels of ALK using immunohistochemistry (IHC), to evaluate the frequency of ALK gene amplifications and copy number changes with fluorescence *in situ* hybridization (FISH), and to elucidate the prevalence, clinicopathological characteristics, and prognostic relevance of ALK gene alterations in IBC.

## Materials and Methods

### Ethics statement

This study was approved by the Institutional Review Board (IRB) of each involved institution: Severance Hospital, Asan Medical Center, National Cancer Center, and Gangnam Severance Hospital. Written informed consents were obtained from all patients for genetic analysis of tumor tissues.

### Patients

Tumor tissues and medical records of IBC patients were retrospectively collected from several institutes in Korea (Severance Hospital, Asan Medical Center, National Cancer Center, and Gangnam Severance Hospital). Patients were diagnosed with invasive ductal or lobular carcinoma of the breast between August 1996 and December 2011, and fulfilled the diagnostic criteria of IBC: diffuse erythema and dermal edema (*peau d’orange*) of a third or more of the skin of the breast, based on the definition of IBC by the American Joint Committee on Cancer (AJCC). Medical records were reviewed in regards to clinical parameters including patient age at initial diagnosis, pathologic tumor stage based on the seventh edition of the AJCC, date of diagnosis, date of recurrence, date of death, treatment modalities that each patient received (surgery type, chemotherapy regimen, radiation therapy, and hormone therapy), and pathologic reports including estrogen receptor (ER), progesterone receptor (PR), and HER2 status. All patients were followed up at each institute until the date of death or the last day of follow up, and the median follow up was 26.2 months (95% confidence interval [CI], 11.8–40.6 months).

### ALK Fluorescence *in situ* hybridization (FISH)

**Fig 1:**
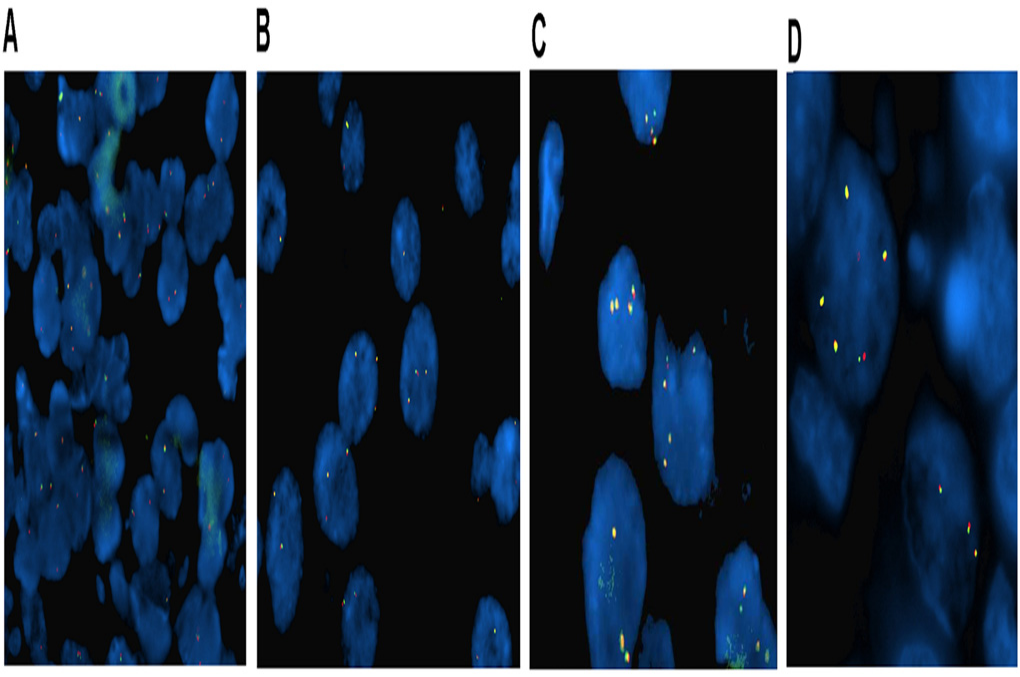
Representative ALK fluorescent *in situ* hybridization (FISH) images in IBC patients. (A) disomy, (B) ALK copy number gain (trisomy), (C) ALK copy number gain (polysomy), and (D) ALK amplification.

We performed FISH analysis in Samkwang Medical Laboratory, which has certification from CAP (College of American Pathologists). To assess the genetic status of ALK, we used an ALK LSI break-apart (2p23) probe (Abbott Molecular Inc., Des Plaines, IL) to detect rearrangements involving the ALK gene and to determine copy numbers. Briefly, representative 4 μm sections of blocks were mounted on slides, air-dried, and baked for 2 hours at 60°C in ThermoBrite. The slides were deparaffinized, dehydrated, immersed in 0.2N HCl, and incubated in 1M NaSCN for 35 minutes at 80°C. Sections were then immersed in protease solution, and the tissues were fixed in 10% neutral-buffered formalin. The slides were incubated in a humidified atmosphere (Hybrite, Vysis) at 73°C for 3 minutes and at 37°C for 19 hours followed by immersion in 0.4 x SSC/0.3% NP-40 at room temperature and at 73°C for 2 minutes. The probe was applied, and the sections were appropriately covered and sealed. After washing and drying, nuclei were counterstained with 4,6-diamidino-2-phenylindole (DAPI). FISH signals were assessed under a Nikon ECLIPSE 80i (Nikon, Japan) equipped with a triple-pass filter (DAPI/Green/Orange; Nikon, Japan). Non-rearranged ALK presented as an overlapping orange/red and green (yellowish) signals. The probes were considered typically rearranged when separated by green and orange/red signals (by at least three times the signal diameter) and were atypically rearranged when a single orange or green signal was observed. We considered the ALK rearrangement to be present if 15% of nuclei had rearranged FISH signal patterns. For ALK copy number status determination, we reviewed previous studies in various tumors [11,13,14,19], and predefined the criteria for ALK gene copy number status prior to the FISH experiment. We arbitrarily classified ALK gene copy number status according to the frequency of tumor cells with specific numbers of copies: trisomy, as 3 fusion signals in ≥30% of cells and ≥4 fusion signals in <10% of cells; polysomy, as ≥4 fusion signals in ≥10% of analyzed cells; and amplification, as ≥6 fusion signals in ≥10% of analyzed cells. An ALK gene copy number below the above criteria thresholds was defined as disomy (Fig. 1). We classified disomy as ALK CNG (-), and trisomy, polysomy, and amplification as ALK CNG (+). The FISH results were interpreted by three independent evaluators (Wooyoung Jung, Yoonjung Kwon, and Yoonmi Seok) who were blinded for clinical data, and any discordance among evaluators was discussed until a consensus was reached.

### Immunohistochemical (IHC) assay

**Fig 2:**
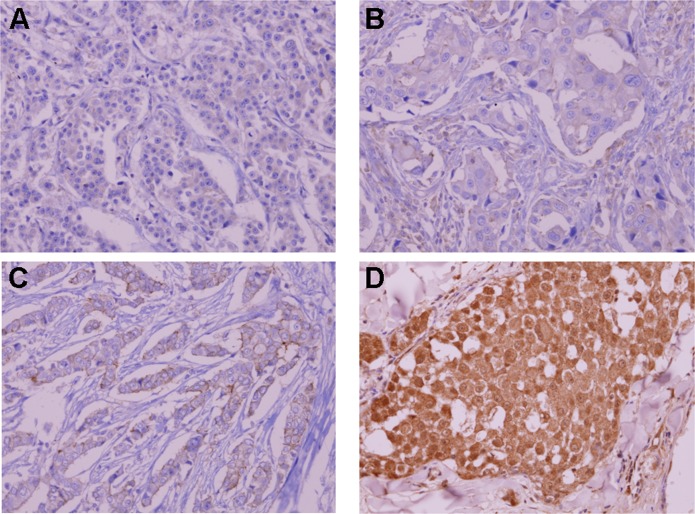
Representative immunohistochemical staining of ALK. (A) negative staining, (B) ≤10% staining, (C) 50% staining, and (D) 80% staining of tumor cells.

We retrieved FFPE tissue specimens of 27 patients for ALK IHC. All archival hematoxylin and eosin (H&E)-stained slides for each patient were reviewed by one pathologist. ALK IHC was performed on 4-μm-thick FFPE tissue specimens using mouse monoclonal antibody for ALK (Invitrogen, 1:200, polyclonal, Fig. 2). ALK staining was scored according to the proportion of stained cells to total analyzed cells. The immunohistochemistry of ER, PR, and HER2 and FISH for HER2 were also conducted by each of the four institutes according to the recommended guidelines of the American Society of Clinical Oncology and College of American Pathologists [20,21].

### Statistical analysis

Data were processed using SPSS for Windows, version 20.0 (SPSS Inc., Chicago, IL, USA). Clinicopathologic characteristics and expression status of ER, PR, and HER2 were compared according to ALK status using either a chi-square test or Fisher’s exact test. Comparisons of the ALK IHC scores with mean ALK copy numbers were performed using Spearman’s rank correlation test. RFS and OS were calculated from the time of initial treatment to the time of event of interest, recurrence after surgery, death, or final clinical follow up. Patient survival was estimated by the Kaplan-Meier method and compared with a log-rank test. Multivariate analysis was performed using a Cox-proportional hazard model to estimate survival with adjustment for factors including AJCC stage, HER2 status, and ER status. Statistical significance was assumed when the p value was less than 0.05; all tests were two-sided.

## Results

### Patient characteristics

Thirty-six IBC patients from four institutes showing HER2-positive disease (44.4%) and TNBC (36.1%) in frequent order were studied. The median age of patients was 51 (range, 29.7–65.2 years), and the median OS was 37.2 months (95% CI, 17.4–57.0 months). Most of the patients had a high clinical node stage (77.8% of patients were higher than N1). Distant metastasis was present at the time of diagnosis in 33.3% of patients (12/36), although no patients had initial brain metastasis. Most stage III patients (n = 22, 91.7%) underwent modified radical mastectomy, and 6 out of 12 stage IV patients also underwent palliative mastectomy after systemic chemotherapy. Among 28 patients who underwent mastectomy, 23 received neoadjuvant chemotherapy before surgery, and 5 patients received adjuvant chemotherapy after surgery. In total, 35 out of 36 (97.2%) patients received chemotherapy, including 7 patients who received palliative chemotherapies without surgery (Table 1). A summary of chemotherapy agents that patients received can be found in S1 Table.

**Table 1:** ALK Copy Number Gain and Baseline Clinicopathological Characteristics.

Characteristic	Total (N = 36)	ALK CNG (-) (N = 19)	ALK CNG (+) (N = 17)	p-value^a^
**Age, years**				
**Median (range)**	51 (29.7~65.2 yrs)	48 (32~64yrs)	51 (28.0~72.0 yrs)	0.744
**Clinical tumor stage**				
**T4d**	36 (100%)	19 (100%)	17 (100%)	
**Clinical node stage**				
**N0**	1 (2.8%)	1 (5.3%)	0 (0%)	0.498
**N1**	7 (19.4%)	4 (21.1%)	3 (17.6%)	
**N2**	8 (22.2%)	4 (21.1%)	4 (23.5%)	
**N3**	20 (55.6%)	10 (52.6%)	10 (58.8%)	
**AJCC stage**				
**stage III**	24 (66.7%)	13 (68.4%)	11 (64.7%)	0.813
**stage IV**	12 (33.3%)	6 (31.6%)	6 (35.3%)	
**Operation**				
**No**	8 (22.2%)	5 (26.3%)	3 (17.6%)	0.695
**Yes**	28 (77.8%)	14 (73.7%)	14 (82.4%)	
**Primary chemotherapy**				
**Neoadjuvant**	23 (63.9%)	11 (57.9%)	12 (70.6%)	1
**Adjuvant**	5 (13.9%)	3 (15.8%)	2 (11.8%)	
**Palliative**	7 (19.4%)	4 (21.1%)	3 (17.6%)	
**None**	1 (2.8%)	1 (5.3%)	0 (0.0%)	
**Use of hormone therapy**				
**No**	26 (72.2%)	13 (68.4%)	13 (76.5%)	0.717
**Yes**	10 (27.8%)	6 (31.6%)	4 (23.5%)	
**Use of Radiation therapy**				
**No**	17 (47.2%)	11 (57.9%)	6 (35.3%)	0.175
**Yes**	19 (52.8%)	8 (42.1%)	11 (64.7%)	
**ER** ^**b**^				
**Negative**	25 (69.4%)	12 (63.2%)	13 (76.5%)	0.387
**Positive**	11 (30.6%)	7 (36.8%)	4 (23.5%)	
**PR** ^**b**^				
**Negative**	28 (77.8%)	14 (73.7%)	14 (82.4%)	0.532
**Positive**	8 (22.2%)	5 (26.3%)	3 (17.6%)	
**HER2**				
**Negative**	20 (55.6%)	10 (52.6%)	10 (58.8%)	0.709
**Positive**	16 (44.4%)	9 (47.4%)	7 (41.2%)	
**Subtype**				
**ER(+)/HER2(-)**	7 (19.4%)	5 (26.3%)	2 (11.8%)	0.468
**ER(+)/HER2(+)**	6 (16.7%)	4 (21.1%)	2 (11.8%)	
**ER(-)/HER2(+)**	10 (27.8%)	5 (26.3%)	5 (29.4%)	
**TNBC**	13 (36.1%)	5 (26.3%)	8 (47.1%)	

ALK, anaplastic lymphoma kinase; CNG, copy number gain; AJCC, American Joint Committee on Cancer; ER, estrogen receptor; PR, progesterone receptor; HER2, human epidermal growth factor receptor 2; TNBC, triple-negative breast cancer.

^a^ *p* value was calculated either by chi-square test or Fisher’s exact test.

^b^All PR positive patients were also ER positive.

### ALK CNG and its correlation with clinicopathological characteristics

ALK CNG (trisomy, polysomy, and amplification) was observed in 17 out of 36 (47.2%) IBC patients; among them, one patient harbored ALK gene amplification. None of the patients had ALK rearrangement in this study. The clinical characteristics of the patients were similar for both the ALK CNG (-) group and the ALK CNG (+) group (Table 1), and received chemotherapy agents were not different between the two groups (S2 Table). There was no significant difference in ER/PR/HER2 status according to ALK CNG, although the proportion of TNBC was higher in ALK CNG (+) patients, without statistical significance (41.2% *vs*. 26.3%, *p* = 0.345). The other tumor subtypes were not relevant to ALK CNG status in this study. All of the new brain metastasis during the follow up period occurred in ALK CNG (+) patients (4/17, 23.5%), and none occurred in ALK CNG (-) patients.

### Immunohistochemical (IHC) of ALK

We performed IHC staining of ALK to correlate gene copy number status and ALK protein expression in 27 patients (Table 2). ALK expression was found in 6 out of 13 ALK CNG (+) IBC tumors, and 9 out of 14 ALK CNG (-) patient tumors. There was no significant correlation between mean ALK gene copy number and ALK IHC score (*p* = 0.767).

**Table 2:** Comparison of ALK Immunohistochemical staining and fluorescence *in situ* hybridization analysis results. (n = 27).

Number	Age	AJCC Stage	ALK^a^≤2F	ALK^a^ 3~4F	ALK^a^≥5F	CNG	ALK IHC Score^b^	Subtype	Survival
1	F/45	III	100%	0%	0%	Negative	0%	TNBC	alive
2	F/41	III	100%	0%	0%	Negative	40%	TNBC	death
3	F/64	III	100%	0%	0%	Negative	80%	ER(-)/HER2(+)	death
4	F/64	IV	98%	2%	0%	Negative	10%	ER(+)/HER2(-)	alive
5	F/72	III	97%	3%	0%	Negative	0%	ER(-)/HER2(+)	alive
6	F/37	IV	97%	3%	0%	Negative	10%	ER(-)/HER2(+)	alive
7	F/52	III	95%	5%	0%	Negative	50%	ER(+)/HER2(+)	death
8	F/53	III	94%	5%	1%	Negative	10%	TNBC	death
9	F/44	IV	94%	6%	0%	Negative	10%	TNBC	death
10	F/45	III	91%	9%	0%	Negative	0%	ER(+)/HER2(+)	alive
11	F/47	III	85%	14%	1%	Negative	0%	ER(-)/HER2(+)	alive
12	F/45	III	83%	17%	0%	Negative	10%	TNBC	alive
13	F/63	IV	83%	17%	0%	Negative	30%	ER(+)/HER2(-)	death
14	F/59	III	75%	25%	0%	Negative	0%	ER(+)/HER2(-)	alive
15	F/63	III	70%	30%	0%	Positive	20%	TNBC	death
16	F/51	IV	69%	31%	0%	Positive	0%	ER(-)/HER2(+)	alive
17	F/64	IV	64%	28%	8%	Positive	0%	TNBC	death
18	F/52	III	60%	40%	0%	Positive	60%	ER(-)/HER2(+)	death
19	F/63	III	57%	41%	2%	Positive	0%	ER(+)/HER2(+)	alive
20	F/51	IV	53%	47%	0%	Positive	0%	TNBC	death
21	F/34	III	53%	47%	0%	Positive	10%	TNBC	death
22	F/55	III	45%	55%	0%	Positive	0%	ER(+)/HER2(+)	death
23	F/56	IV	44%	49%	7%	Positive	0%	ER(-)/HER2(+)	death
24	F/59	III	38%	18%	44%	Amplification	10%	ER(+)/HER2(-)	death
25	F/28	III	33%	57%	10%	Positive	80%	TNBC	death
26	F/43	III	23%	50%	27%	Positive	80%	TNBC	death
27	F/54	IV	11%	70%	19%	Positive	0%	TNBC	death

AJCC, American Joint Committee on Cancer; ALK, anaplastic lymphoma kinase; CNG, copy number gain; IHC, immunohistochemical staining; ER, estrogen receptor; PR, progesterone receptor; HER2, human epidermal growth factor receptor 2; TNBC, triple-negative breast cancer.

^a^ALK gene copy number status according to the frequency of tumor cells with specific numbers of copies in fluorescence *in situ* hybridization.

^b^ALK IHC score according to the proportion of stained cells to total analyzed cells

### ALK CNG status and patient survival

**Fig 3:**
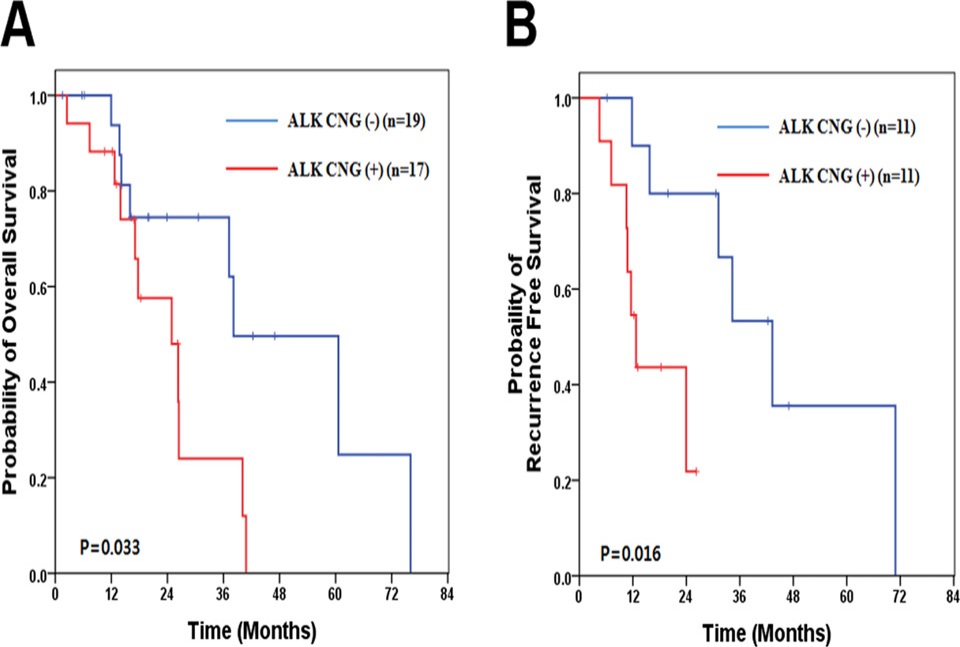
Comparison of overall survival and recurrence free survival after mastectomy in ALK CNG (-) patients and ALK CNG (+) patients. (A) overall survival and (B) recurrence free survival. ALK, anaplastic lymphoma kinase; CNG, copy number gain.

In univariate analysis, median OS was significantly worse in ALK CNG (+) than in ALK CNG (-) (24.9 months *vs*. 38.1 months, *p* = 0.033, Fig. 3). For stage III patients who underwent modified radical mastectomy (n = 22), RFS was also significantly shorter in ALK CNG (+) patients than in ALK CNG (-) patients. (12.7 months *vs*. 43.3 months, *p* = 0.016). We also performed subgroup OS analysis for both stage III (n = 24) and stage IV (n = 12) subgroups. There was a tendency of shorter OS in ALK CNG (+) patients compared to ALK CNG (-) patients in the stage III subgroup (26.3 months vs. 60.6 months, *p* = 0.058, S1 Fig.), however, it was not statistically significant. There was no OS difference between ALK CNG (+) and ALK CNG (-) patients in the stage IV subgroup (*p* = 0.574). Comparison of progression-free survival was not performed for metastatic IBC due to the small sample size (n = 12). Multivariate analysis of OS and RFS using the Cox-proportional hazard model was performed with adjustment for several factors (AJCC stage, ER status, and HER2 status for OS; ER status and HER2 status for RFS). Multivariate analysis showed significantly worse RFS for ALK CNG (+) patients than for ANK CNG (-) patients (HR 5.63, 95% CI 1.11–28.44, *p* = 0.037). There was also a tendency of worse OS in ALK CNG (+) patients in multivariate analysis (HR 2.63, 95% CI 0.86–8.11, *p* = 0.076, Table 3).

**Table 3:** Analyses of Prognostic Factors for Overall Survival and Recurrence-free Survival. (Cox-Proportional Harzard Model).

	Overall Survival (n = 36)		Recurrence-Free Survival (n = 22)	
Factors	Hazard ratio (95% CI)	P- value^a^	Hazard ratio (95% CI)	P- value*
ALK CNG (+) *vs*. CNG(-)	2.63 (0.86–8.11)	0.076	5.63 (1.11–28.44)	0.037
ER positive *vs*. negative	0.67 (0.21–2.16)	0.504	0.78 (0.23–2.65)	0.694
HER2 positive *vs*. negative	0.63 (0.21–1.89)	0.404	0.77 (0.23–2.51)	0.660
AJCC stage IV *vs*. III	2.74 (0.86–8.73)	0.671		

ALK, anaplastic lymphoma kinase; CNG, copy number gain; AJCC, American Joint Committee on Cancer; ER, estrogen receptor; HER2, human epidermal growth factor receptor 2.

^a^ *p* values were calculated using the Cox-proportional hazard model.

## Discussion

Therapeutic targeting of ALK rearrangement has led to a remarkable improvement of survival in ALK-rearranged NSCLC patients [10,22], and many studies are underway to reveal the oncogenic role of ALK in other tumor species. Beyond ALK gene rearrangement, ALK gene aberrations such as mutations, copy number changes, and amplifications have also been reported in various malignancies, and their clinical implication and susceptibility to ALK inhibitors are attracting interest amongst researchers. ALK gene fusion occurs in limited subsets of cancer such as NSCLC, anaplastic large cell lymphoma, and inflammatory myofibroblastic tumor, whereas ALK mutation and copy number gain were observed in a wide range of cancers.

We evaluated the prevalence of ALK gene expression and copy number change, as well as their relationships to clinical characteristics and prognosis in IBC patients. We found frequent copy number gains of the ALK gene (47.2%) in FISH analysis, and there was also a presence of ALK protein expressions (55.5%) in IBC tumors, found in IHC analysis. However, ALK CNG was not correlated with ALK protein expression level in this study. ALK CNG positivity was independently associated with significantly poorer RFS in survival analysis. Based on our finding, we suggest that ALK CNG may play a role in IBC pathogenesis and contribute to poor prognosis of IBC patients. Since many IBC patients receive standard chemotherapy that is similar to non-IBC patients without specific molecular targets, the validation of the relationship between ALK CNG and susceptibility to ALK inhibitor calls for further investigation.

Previous studies did not observe ALK rearrangement in breast cancer patients [23,24]. However, Perez-Pinera et al. demonstrated the expression of ALK in different histological subtypes of human breast cancer [25], and TCGA (The Cancer Genome Atlas Network) genomic analysis showed ALK gene copy number gains in 43 out of 476 breast cancer patients [26]. Importantly, recent studies have reported frequent ALK CNGs in IBC tumors, with a frequency of 64%–80%, and a preclinical study also found ALK CNG in IBC cell lines and induction of apoptosis and cell death by crizotinib treatment [16,18]. Although such studies only tested limited sample size without direct comparison between IBC tumors and non-IBC tumors [16, 18], ALK has been suggested as a potential druggable target in IBC patients, and its clinical implication and prognostic significance needs to be elucidated.

Interestingly, recent studies also reported discordance between ALK CNG (or amplification) on FISH and ALK protein expression on IHC in various tumor tissues [12,18,27–29], and ALK IHC results were similarly not correlated with ALK CNG in the present study. Although we did not study the mechanism further, epigenetic regulations and post-transcriptional silencing may be possible explanations for discordance between ALK CNG and ALK IHC. In addition, we cannot rule out the possibility that the ALK antibody that we used in the current study was not sensitive enough to detect ALK protein level change accompanied by ALK CNG. Although ALK copy number status is not well correlated with ALK IHC, ALK CNG has been associated with poor prognosis in several malignancies [12,14,30]. Robertson et al. also showed activation of ALK and its downstream signaling pathways in ALK-amplified IBC cells, as well as *in vivo* functional relevance of ALK amplification in an IBC xenograft model [16]. Further functional studies are required that investigate the molecular mechanisms of ALK CNG upon cancer pathogenesis and the relationship between ALK CNG and ALK protein expression level.

In this study, we used the FDA-approved FISH probe to evaluate ALK gene status in tumors. In previous studies, the cutoffs of ALK gene copy number gain in FISH have been defined differently in various malignancies [11–14,16,19]. Considering that low-level CNG may simply reflect DNA duplication of dividing tumor cells, we predefined the cutoff of tumor cell percentage for trisomy as ≥30%, and polysomy was defined as ≥4 fusion signals in ≥10% to avoid overestimation of CNG. However, the criteria for ALK CNG determination need to be refined by further studies.

In this study, the proportion of TNBC was higher in ALK CNG (+) patients (47.1%) than in ALK CNG (-) (26.3%). Although statistical significance was not reached probably due to the small sample size, this result suggests that ALK CNG is common in TNBC among four subtypes of breast cancer. Lehmann et al. have also presented enriched genetic aberrations in ALK pathways in mesenchymal-type TNBCs [31]. As TNBCs have the worst prognosis and do not yet have known molecular targets, ALK could provide a potential druggable target especially for triple-negative IBC.

There are limited numbers of studies on ALK CNG and survival outcome, and not much has been studied on the relationship between ALK CNG and tumor behavior as yet. However, ALK CNG has been related to a higher rate of metastatic disease and poor survival in rhabdomyosarcoma and colorectal carcinomas [12,14]. Additionally, Kim et al. reported higher ALK CNG and protein expression in metastatic lesions compared to primary tumors in NSCLC patients [32]. Our study demonstrated significantly worse RFS for ALK CNG (+) IBC patients than for ALK CNG (-) patients, and we suggest that ALK CNG may play a role in IBC progression and metastasis based on these findings. However, OS was not significantly different according to ALK CNG status with our limited sample size and follow up duration, although there was a tendency of worse OS in ALK CNG (+) patients in multivariate analysis. It is imperative to study the prognostic significance of ALK CNG further in other populations with a larger sample size and longer follow up period.

Although it seems evident that copy number changes of the ALK gene are prevalent in IBC, the association between high copy number status of ALK and favorable response to ALK inhibitors remains undetermined. Several reports have shown effective growth inhibition by crizotinib in neuroblastoma, NSCLC, and IBC cell lines harboring ALK copy number gain or amplification in preclinical studies [16,33,34]. These findings support the possible therapeutic potency of ALK inhibitors in the treatment of tumors containing ALK gene copy number gain. Selective ALK inhibitors are currently under clinical trial in patients with advanced human solid tumors containing ALK gene alterations. The clinical efficacy of these inhibitors in ALK high copied or amplified IBC patients is yet to be known. Results from early phase clinical trials will help future studies to focus on the oncogenic role of ALK in IBC pathogenesis and the potential effect of ALK inhibitors in IBCs.

There are several caveats to this study. First, we evaluated only Korean IBC patients. Second, the follow up duration of this study was relatively short, and may have limited sufficient comparison of OS between ALK CNG (+) and ALK CNG (-) groups. Third, all data and specimens were achieved retrospectively, and we used only a single technique to measure ALK CNG. Finally, the FISH results were interpreted in a single laboratory, although three independent evaluators participated. Therefore, a larger study in an independent population of a different ethnic background with comprehensive analysis of ALK copy number status is warranted. However, we believe that this is the first study to observe the clinical significance of ALK gene alterations in IBC.

In summary, this study showed significantly frequent ALK CNG in IBC patients, and ALK CNG was associated with poorer RFS in curatively resected patients. Our finding suggests that ALK CNG may have prognostic significance in IBC patients, and it is necessary to explore its susceptibility to ALK inhibitors.

## Supporting Information

S1 Fig Subgroup analysis comparing overall survival according to ALK copy number status. (A) in stage III subgroup (n = 24), and (B) in stage IV subgroup (n = 12).
(TIF)

S1 Table Patient list including chemotherapy agents received. (DOCX)

S2 Table Comparison of used chemotherapy agents according to ALK Copy Number Gain. (DOCX)
