# Supplementary figures and images for: Anaplastic Lymphoma Kinase Gene Copy Number Gain in Inflammatory Breast Cancer (IBC): Prevalence, Clinicopathologic Features and Prognostic Implication
Source: PLoS One. 2015 Mar 24;10(3):e0120320. doi: 10.1371/journal.pone.0120320 (PMC4372579; doi:10.1371/journal.pone.0120320)

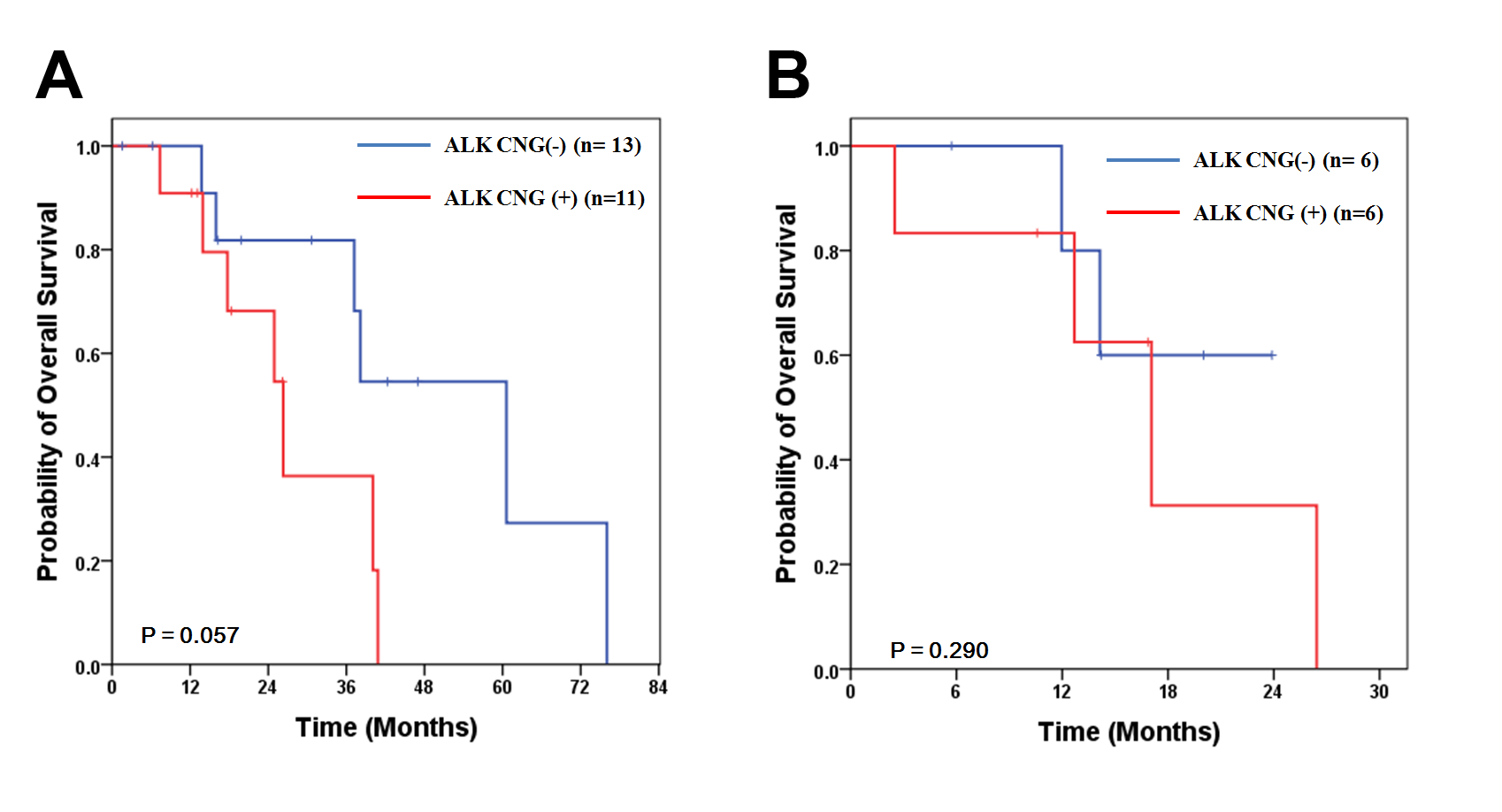

Supplement: S1 Fig — (A) in stage III subgroup (n = 24), and (B) in stage IV subgroup (n = 12). (TIF) [file pone.0120320.s001.tif]
