# Supplementary material for: Anaplastic Lymphoma Kinase Gene Copy Number Gain in Inflammatory Breast Cancer (IBC): Prevalence, Clinicopathologic Features and Prognostic Implication
Source: PLoS One. 2015 Mar 24;10(3):e0120320. doi: 10.1371/journal.pone.0120320 (PMC4372579; doi:10.1371/journal.pone.0120320)
Supplement: S1 Table — (DOCX) [file pone.0120320.s002.docx]

**S1 Table. Patient list including chemotherapy agents received**

| **Sex /Age** | **Stage** | **ALK CNG** | **Subtype** | **Primary Chemotherapy^a^** | **Use of HER2 targeting agent^b^** | **Operation** | **Recurrence** | **Survival** | **Used palliative chemotherapy agent** |
| --- | --- | --- | --- | --- | --- | --- | --- | --- | --- |
| F/54 | IV | Positive | TNBC | OP+ Adjuvant CMF | No | Yes | Yes | Death | Paclitaxel/Ifosphamide |
| F/59 | III | Negative | ER(+)/HER2(-) | Neoadjuvant AT + OP + Adjuvant AT | No | Yes | No | Alive |  |
| F/47 | III | Negative | ER(-)/HER2(+) | Neoadjuvant FAC + OP + Adjuvant FAC/Trastuzumab | Yes | Yes | No | Alive |  |
| F/64 | IV | Positive | TNBC | Neoadjuvant AT + OP | No | Yes | Yes | Death | Capecitabine, Gemcitabine/ Cisplatin, Paclitaxel/Ifosphamide, Navelbine, Ixabepilone |
| F/72 | III | Negative | ER(-)/HER2(+) | OP + Adjuvant CMF | No | Yes | No | Alive |  |
| F/56 | IV | Positive | ER(-)/HER2(+) | Palliative Chemo (Trastuzumab/Paclitaxel/Everolimus) | Yes | No | N/A**^c^** | Death | Trastuzumab/Paclitaxel/Everolimus |
| F/63 | III | Positive | ER(+)/HER2(+) | Neoadjuvant AC/Docetaxel + OP + Adjuvant Trastuzumab | Yes | Yes | No | Alive |  |
| F/51 | IV | Positive | ER(-)/HER2(+) | Palliative Chemo (Trastuzumab/Paclitaxel) | Yes | No | N/A | Alive | Trastuzumab/Paclitaxel |
| F/37 | IV | Negative | ER(-)/HER2(+) | Neoadjuvant FAC + OP | Yes | Yes | Yes | Alive | Trastuzumab/Paclitaxel, Lapatinib/Capecitabine,  Vinorelbine/Cisplatin |
| F/51 | IV | Positive | TNBC | Palliative Chemo (Gemcitabine/Cisplatin) | No | No | N/A | Death | Gemcitabine/Cisplatin, Doxorubicin/Cyclophosphamide |
| F/53 | III | Negative | TNBC | Palliative Chemo (AC) | No | No | N/A | Death | Doxorubicin/Cyclophosphamide, Paclitaxel/Cisplatin, Capecitabine |
| F/45 | III | Negative | TNBC | OP + adjuvant AC | No | Yes | No | Alive |  |
| F/45 | III | Negative | ER(+)/HER2(+) | No Treatment | No | No | N/A | Alive |  |
| F/45 | III | Negative | TNBC | Neoadjuvant AC + OP | No | Yes | No | Alive |  |
| F/64 | IV | Negative | ER(+)/HER2(-) | Palliative Chemo (Paclitaxel) | No | No | N/A | Alive | Paclitaxel |
| F/32 | III | Positive | ER(+)/HER2(-) | Neoadjuvant AT + OP | No | Yes | No | Alive |  |
| F/32 | IV | Negative | ER(+)/HER2(-) | Neoadjuvant AT + OP | No | Yes | No | Alive |  |
| F/49 | III | Negative | ER(-)/HER2(+) | Neoadjuvant AC + OP -> Adjuvant Trastuzumab | Yes | Yes | Yes | Alive | Trastuzumab/Paclitaxel, Capectiabine/Lapatinib |
| F/56 | IV | Negative | ER(+)/HER2(+) | Palliative Chemo (Doxorubicin/Docetaxel) | Yes | No | N/A | Alive | Doxorubicin/Docetaxel, Trastuzumab |
| F/51 | III | Positive | ER(-)/HER2(+) | Neoadjuvant AC + OP + Adjuvant Trastuzumab | Yes | Yes | No | Alive |  |
| F/30 | III | Positive | ER(-)/HER2(+) | Neoadjuvant AC + Neoadjuvant TCH + OP | Yes | Yes | No | Alive |  |
| F/48 | IV | Positive | TNBC | Neoadjuvant AC + Trastuzumab/Paclitaxel + OP | Yes | Yes | No | Alive |  |
| F/42 | III | Negative | ER(+)/HER2(+) | Neoadjuvant FAC + OP | No | Yes | Yes | Death | Capecitabine, Navelbine |
| F/43 | III | Positive | TNBC | Neoadjuvant AT + OP | No | Yes | Yes | Death |  |
| F/63 | III | Positive | TNBC | Neoadjuvant AT + OP + Adjuvant AT | No | Yes | Yes | Death |  |
| F/48 | III | Negative | ER(+)/HER2(-) | Neoadjuvant AT + OP | No | Yes | Yes | Death |  |
| F/28 | III | Positive | TNBC | Neoadjuvant FAC + OP | No | Yes | Yes | Death |  |
| F/55 | III | Positive | ER(+)/HER2(+) | Neoadjuvant AC + OP + Adjuvant Docetaxel | Yes | Yes | Yes | Death | Trastuzumab/Paclitaxel |
| F/34 | III | Positive | TNBC | Neoadjuvant Capecitabine/Vinorebine + OP  + Adjuvant Docetaxel | No | Yes | Yes | Death |  |
| F/52 | III | Negative | ER(+)/HER2(+) | Neoadjuvant AT + OP | No | Yes | Yes | Death |  |
| F/52 | III | Positive | ER(-)/HER2(+) | Neoadjuvant FAC + OP | Yes | Yes | Yes | Death | Trastuzumab/Paclitaxel |
| F/63 | IV | Negative | ER(+)/HER2(-) | Palliative Chemo (FEC) | No | No | N/A | Death |  |
| F/59 | III | Positive | ER(+)/HER2(-) | OP + Adjuvant FEC | No | Yes | Yes | Death |  |
| F/44 | IV | Negative | TNBC | Neoadjuvant FEC + OP | No | Yes | Yes | Death | Docetaxel |
| F/64 | III | Negative | ER(-)/HER2(+) | OP + Adjuvant CMF | No | Yes | Yes | Death |  |
| F/41 | III | Negative | TNBC | Neoadjuvant AT + OP | No | Yes | Yes | Death | Doxifluridine |

ALK, anaplastic lymphoma kinase; CNG, copy number gain; ER, estrogen receptor; PR, progesterone receptor; HER2, human epidermal growth factor receptor 2; TNBC, triple-negative breast cancer; OP, Operation.

^a^Chemotherapy regimen abbreviations: AC, Doxorubicin + Cyclophosphamide; AT, Doxorubicin + Docetaxel; CMF, Cyclophosphamide + Methotrexate + Fluorouracil; FAC, Fluorouracil + Doxorubicin + Cyclophosphamide; FEC, Fluorouracil + Epirubicin + Cyclophosphamide; TCH, Docetaxel + Carboplatin + Trastuzumab

^b^Use of HER2 targeting chemotherapy agents including trastuzumab and lapatinib during whole course of treatment after diagnosis

^c^Not applicable for patients who did not receive operation.
