# Supplementary material for: Anaplastic Lymphoma Kinase Gene Copy Number Gain in Inflammatory Breast Cancer (IBC): Prevalence, Clinicopathologic Features and Prognostic Implication
Source: PLoS One. 2015 Mar 24;10(3):e0120320. doi: 10.1371/journal.pone.0120320 (PMC4372579; doi:10.1371/journal.pone.0120320)
Supplement: S2 Table — (DOCX) [file pone.0120320.s003.docx]

**S2 Table. Comparison of used chemotherapy agents according to ALK Copy Number Gain**

|  | **Total** | **ALK CNG (-)** | **ALK CNG (+)** |  |
| --- | --- | --- | --- | --- |
|  | **N=36** | **N=19** | **N=17** | **p-value**^a^ |
| **Primary chemotherapy^b^** |  |  |  |  |
| **Neoadjuvant** | 23 (63.9%) | 11 (57.9%) | 12 (70.6%) |  |
| **AC** | 6 (26.1%) | 2 (18.2%) | 4 (33.3%) | 0.453 |
| **AT** | 9 (39.1%) | 5 (45.5%) | 4 (33.3%) |  |
| **FAC or FEC** | 6 (26.1%) | 4 (36.4%) | 2 (16.7%) |  |
| **Others** | 2 (8.7%) | 0 (0.0%) | 2 (16.7%) |  |
|  |  |  |  |  |
| **Adjuvant** | 5 (13.9%) | 3 (15.8%) | 2 (11.8%) |  |
| **AC** | 1 (20.0%) | 1 (66.7%) | 0 (0.0%) | 1.000 |
| **CMF** | 3 (60.0%) | 2 (33.3%) | 1 (50%) |  |
| **FEC** | 1 (20.0%) | 0 (0.0%) | 1 (50%) |  |
|  |  |  |  |  |
| **Palliative** | 7 (19.4%) | 4 (21.1%) | 3 (17.6%) |  |
| **None** | 1 (2.8%) | 1 (5.3%) | 0 (0.0%) |  |
|  |  |  |  |  |
| **Use of HER2 targeting agents** |  |  |  |  |
| **No** | 24 (66.7%) | 15 (78.9%) | 9 (52.9%) | 0.098 |
| **Yes** | 12 (33.3%) | 4 (21.1%) | 8 (47.1%) |  |

ALK, anaplastic lymphoma kinase; CNG, copy number gain; ER, estrogen receptor; PR, progesterone receptor; HER2, human epidermal growth factor receptor 2; TNBC, triple-negative breast cancer.

^a^ *p* value was calculated either by chi-squared test or Fisher’s exact test.

^b^Chemotherapy regimen abbreviations: AC, Doxorubicin + Cyclophosphamide; AT, Doxorubicin + Docetaxel; FAC, Fluorouracil + Doxorubicin + Cyclophosphamide; FEC, Fluorouracil + Epirubicin + Cyclophosphamide; CMF, Cyclophosphamide + Methotrexate + Fluorouracil.
